# Discovery of a Novel Induced Polymorphism in *SD*1 Gene Governing Semi-Dwarfism in Rice and Development of a Functional Marker for Marker-Assisted Selection

**DOI:** 10.3390/plants9091198

**Published:** 2020-09-14

**Authors:** Shivashankar Bhuvaneswari, Subbaiyan Gopala Krishnan, Ranjith Kumar Ellur, Kunnummal Kurungara Vinod, Haritha Bollinedi, Prolay Kumar Bhowmick, Vijay Prakash Bansal, Mariappan Nagarajan, Ashok Kumar Singh

**Affiliations:** 1 Division of Genetics, ICAR-Indian Agricultural Research Institute, New Delhi 110012, Delhi, India; bhuvana0284@gmail.com (S.B.); krish.icar@gmail.com (S.G.K.); ranjithellur@gmail.com (R.K.E.); kkvinod@iari.res.in (K.K.V.); haritha.agrico@gmail.com (H.B.); prolaybhowmick@gmail.com (P.K.B.); bansal.vijay0@gmail.com (V.P.B.); 2 Division of Plant Breeding, ICAR RC NEH Region, Manipur Centre, Imphal 795004, Manipur, India; 3 Rice Breeding and Genetics Research Centre, ICAR- Indian Agricultural Research Institute, Aduthurai 612101, Tamil Nadu, India; drmnagarajan2000@yahoo.co.in

**Keywords:** rice, semi-dwarfism, mutation, novel SNP, *sd1-bm* allele, dCAPS marker

## Abstract

The semi-dwarfing allele, *sd1-d,* has been widely utilized in developing high-yielding rice cultivars across the world. Originally identified from the rice cultivar Dee-Geo-Woo-Gen (DGWG), *sd1-d*, derived from a spontaneous mutation, has a 383-bp deletion in the *SD1* gene. To date, as many as seven alleles of the *SD1* gene have been identified and used in rice improvement, either with a functional single-nucleotide polymorphism (SNP), with insertion–deletions (InDels), or both. Here, we report discovery of a novel SNP in the *SD1* gene from the rice genotype, Pusa 1652. Genetic analysis revealed that the inheritance of the semi-dwarfism in Pusa 1652 is monogenic and recessive, but it did not carry the *sd1-d* allele. However, response to exogenous gibberellic acid (GA_3_) application and the subsequent bulked segregant and linkage analyses confirmed that the *SD1* gene is involved in the plant height reduction in Pusa 1652. Sequencing of the *SD1* gene from Pusa 1652 revealed a novel transition in exon 3 (T/A) causing a nonsense mutation at the 300th codon. The stop codon leads to premature termination, resulting in a truncated protein of *OsGA20ox2* obstructing the GA_3_ biosynthesis pathway. This novel recessive allele, named *sd1-bm*, is derived from Bindli Mutant 34 (BM34), a γ-ray induced mutant of a short-grain aromatic landrace, Bindli. BM34 is the parent of an aromatic semi-dwarf cultivar, Pusa 1176, from which Pusa 1652 is derived. The semi-dwarfing allele, *sd1-bm,* was further validated by developing a derived cleaved amplified polymorphic sequence (dCAPS) marker, AKS-sd1. This allele provides an alternative to the most widely used *sd1-d* in rice improvement programs and the functional dCAPS marker will facilitate marker-assisted introgression of the semi-dwarf trait into tall genotypes.

## 1. Introduction

The semi-dwarf genes in rice and wheat that spurred a ‘green revolution’ during the 1960s are the most utilized genes in modern plant breeding. Compared to traditional tall varieties, the shortened culm imparted by these genes has an improved lodging resistance, harvest index, nutrient use efficiency and yield in modern cultivars. As per the recent reports, at least 76 plant height mutants and 85 genes have been identified in rice [1,2]. However, barring the phenomenal success of a semi-dwarf allele, *sd1-d* from the Taiwanese cultivar Dee-Geo-Woo-Gen (DGWG), which led to the green revolution in rice, no other dwarfing gene has found to have such a wide utility in rice improvement programs. This is mainly due to these genes’ adverse phenotypic effects, including severe stunting, poor fertility, shorter grain size, etc. [3]. These plant height reduction genes are mostly found to be associated with the regulation or biosynthesis of phytohormones such as gibberellic acid (GA_3_) [4,5], brassinosteroids [6] and strigolactones [7]. Of these, genes involved in GA metabolism are the most studied and widely used [1,2,4].

In rice, semi-dwarf plant height results from the impairment of endogenous GA_3_ production. There are six genes involved in the GA_3_ biosynthesis pathway, namely *ent*-copalyl diphosphate synthase 1 (*OsCPS1*), *ent*-kaurene synthase 1 (*OsKS1*), *ent*-kaurene oxidase 2 (*OsKO2*), *ent*-kaurene oxidase (*OsKAO*), gibberellic acid 20-oxidase 2 (*OsGA20ox2*), and gibberellic acid 3 beta-hydroxylase 2 (*OsGA3ox2*) [4]. Theoretically, a functional mutation in any of these genes could result in defective GA_3_ biosynthesis, leading to semi-dwarfism. However, almost all of the reported semi-dwarf mutations are limited to a single gene, *OsGA20ox2*, popularly known as *SD1*. The *SD1* gene is located on the long arm of chromosome 1 and possesses a 3123-bp-long open reading frame (ORF) carrying three exons and two introns. Exons 1, 2 and 3 are, respectively, 557-, 321- and 291-bp long, interspersed with introns 103 and 1472 bp in length. *SD1* encodes GA20ox2, a 389-amino acid-long enzyme involved in the catalytic conversion of GA12/GA53 to bioactive GA precursors GA9/GA20. Expression of *OsGA20ox2* is found to occur in leaf blades, stems and unopened flowers [4,8,9].

The *sd1-d* allele from DGWG is a mutant allele of the *SD1* functional gene, *OsGA20ox2*, which is characterized by a 383-bp deletion starting from the middle of exon 1 and extending to the upstream of exon 2. Originating spontaneously, this deletion results in a frameshift, leading to polypeptide truncation [10]. When transferred to *indica* cultivars, the *sd1-d* allele reduces the plant height without any adverse effect on plant growth and reproduction, but also increases the yield through the production of a greater number of productive tillers and more grains per panicle. The *sd1-d* gene has been extensively used in the development of improved *indica* rice varieties, almost throughout south and southeast Asia [8,11]. In contrast, a few other *SD1* alleles such as the Jukkoku allele, Calrose 76 allele and Reimei allele have been used in *japonica* cultivar development [12]. In the present study, we report the discovery of a novel *SD1* allele in the short-grain aromatic rice genotype Pusa 1652, its causal mutation and the development of a functional marker.

## 2. Results

### 2.1. Monogenic Inheritance of Plant Height

**Figure 1:**
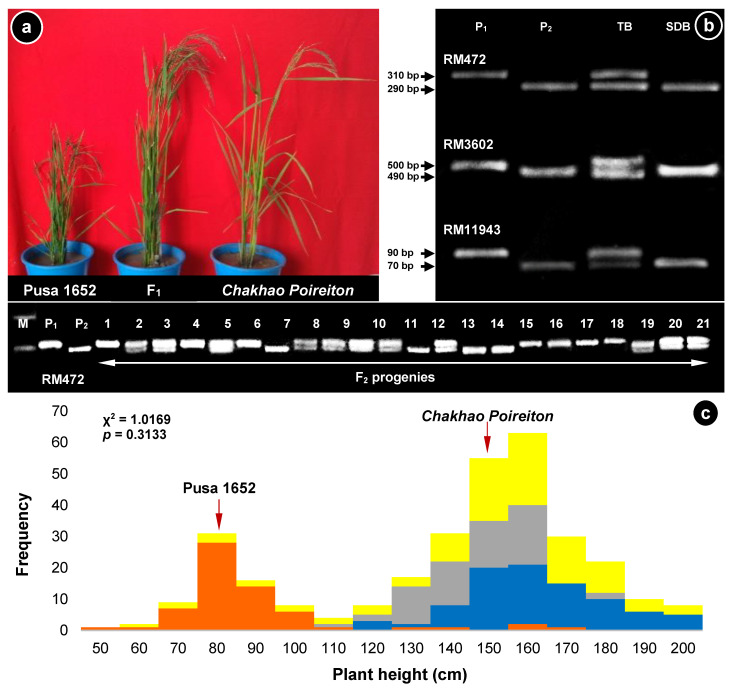
(**a**) Representative photograph showing the differences in plant height of the parents, Pusa 1652 (semi-dwarf), F_1_ (tall) and *Chakhao Poireiton* (tall); (**b**) amplification profile of three putatively linked markers identified through Bulked Segregant Analysis. P_1_—*Chakhao Poireiton*, P_2_—Pusa 1652, TB—tall bulk, SDB—semi-dwarf bulk. TB and SDB represent 10 tall plants and 10 semi-dwarf plants, respectively, in the F_2_ population from the cross, Pusa 1652/*Chakhao Poireiton*; (**c**) frequency distribution of plant height among the F_2_ segregants possessing Pusa 1652 allele (orange), *Chakhao Poireiton* allele (blue) and heterozygotes (grey) based on the RM472 marker. The yellow bars indicate the overall frequency distribution of F_2_ plants. A representative amplification profile of RM472 in the F_2_ generation is placed above the graph, M indicates the 100 bp ladder.

The average plant heights of the parents, *Chakhao Poireiton* and Pusa 1652, was 154.6 and 85.5 cm, respectively, categorizing the parents as tall and semi-dwarf. Based on a paired t-test, the mean height of ten F_1_ plants (155.9 cm) was found to be statistically on par with the tall parent, *Chakhao Poireiton* (Figure 1). In the F_2_ generation, there were two predominant classes of tall and semi-dwarf plants. The distribution of plant height among the 315 F_2_ plants showed a clear bimodal distribution with 244 tall plants and 71 semi-dwarf plants with a division point around 110 cm. The segregation for plant height showed a good fit, with a 3:1 ratio (χ^2^ value of 1.02) between tall and semi-dwarf classes and a *p*-value of 0.313, suggesting that the inheritance of plant height in Pusa 1652 is monogenic.

### 2.2. GA_3_ Response at the Seedling Stage

To confirm whether the semi-dwarfism of Pusa 1652 is due to reduced synthesis of endogenous GA_3_, exogenous application of GA_3_ was carried out. The mean seedling height before and after GA_3_ application varied significantly in all the genotypes, Pusa 1652, IR64 and *Chakhao Poireiton* (Table 1; Figure S1). The effect of exogenous GA_3_ treatment across the genotypes was computed as the relative response of the absolute plant height 18 days after sowing. It was found that both Pusa 1652 and the *sd1-d* check, IR64, responded equally well to GA_3_ application, by showing similar response estimates for absolute plant height. By comparing the relative response, we could eliminate the genotypic differences in seedling plant height, both at the initial stage and after GA_3_ treatment. It was found that GA_3_ spray resulted in a significantly higher seedling elongation response (41.4 % in IR64 and 45.3% in Pusa 1652), compared to 22.4% in *Chakhao Poireiton*, indicating that semi-dwarfism of Pusa 1652 is associated with endogenous GA_3_ production.

### 2.3. Delineating the Involvement of SD1 Locus

The *sd1-d* functional marker amplified a fragment of 731 bp in tall genotypes, *Chakhao Poireiton* and Nagina 22, as well as in semi-dwarf genotypes, Pusa 1652 and its parent, Pusa 1176. However, the amplicon in semi-dwarf rice varieties, IR64 and Pusa Basmati 1, possessing the *sd1-d* allele, was 348 bp. These results confirmed the absence of the *sd1-d* allele in Pusa 1652 and Pusa 1176. A polymorphism survey between the parents of the cross, Pusa 1652/*Chakhao Poireiton*, with 12 Simple Sequence Repeat (SSR) markers flanking the *SD1* region on chromosome 1, identified eight polymorphic markers between *Chakhao Poireiton* and Pusa 1652 (Figure S2). Bulked segregant analysis (BSA) using these eight polymorphic markers in the tall and semi-dwarf bulks derived from the F_2_ segregants identified three SSR markers, namely RM472, RM11943, and RM3602, showing putative co-segregation with plant height (Figure 1b). Among these, RM472 was selected for further genotyping, because of its relatively closer proximity to *SD1*, as well as the better resolution of the alleles due to the larger amplicon size difference. RM472 amplified a 310-bp fragment in *Chakhao Poireiton* (tall), and a 290-bp fragment in Pusa 1652 (semi-dwarf). Genotyping of 315 F_2_ plants using RM472 identified 92 segregants with a Pusa 1652 allele, 159 heterozygotes and 64 possessing a *Chakhao Poireiton* allele that clearly differentiated the corresponding height classes (Figure 1c). Linkage analysis with RM472 indicated that the gene governing semi-dwarfism in Pusa 1652 is located 8.7 cM away. Furthermore, single-marker analysis revealed that it explained 71.6% of the phenotypic variation in plant height in the F_2_ population.

### 2.4. SD1 Gene Sequence Analysis

**Figure 2:**
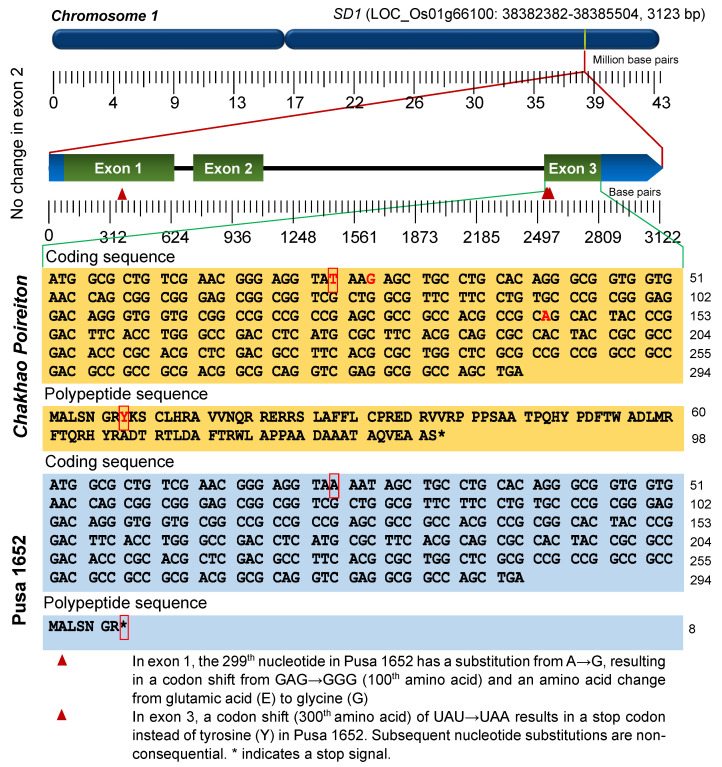
Variations in the amino acid sequence of the SD1 protein from *Chakhao Poireiton* and Pusa 1652. Exon 1 had an amino acid substitution at the 100th position, which changed glutamic acid to glycine in Pusa 1652. There was no variation observed in exon 2, while exon 3 had a stop codon introduced at the 300th amino acid position in Pusa 1652, resulting in the premature termination of GA20 oxidase 2 in exon 3.

The alignment of *SD1* sequences of *Chakhao Poireiton*, Pusa 1652 and Nipponbare (LOC_Os01g66100.1 of IRGSP 1.0 Release 7) could detect four single-nucleotide polymorphisms (SNPs) within the coding region (Figure S3). One of the SNPs, an A→G shift at the physical position 38382764 bp between Nipponbare and Pusa 1652 in exon 1 resulted in an amino acid substitution from glutamic acid to glycine at the 100th amino acid. The remaining three SNPs were detected in exon 3 at positions 38384938 bp (T→A), 38384941 bp (G→T) and 38385057 bp (A→G). Of these, the first SNP resulted in a non-synonymous substitution at the 300th amino acid, leading to a change from tyrosine (TAT) to a stop codon (TAA). The next SNP resulted in an amino acid substitution in the 301st amino acid from lysine to asparagine, while the SNP at 38385057 caused the 340th amino acid to change from glutamine to arginine (Table S1). Therefore, the transversion at 38384938 bp could have resulted in the semi-dwarfism of Pusa 1652 due to the production of a truncated GA20ox2, while the two subsequent SNPs were of little consequence as they were preceded by the stop codon (Figure 2). This polymorphism was reconfirmed by resequencing the PCR amplicon from Pusa 1652 and *Chakhao Poireiton* using a primer pair (forward 5′-ctcctcctcgttggatgtgt-3′, Reverse 5′-gcttctgttcgttccgtttc-3′) covering the genomic region of 38384448-38385242 bp on chromosome 1. This novel semi-dwarfism allele of *SD1*, due to a base substitution (T/A) at positions 38384938 bp in exon 3, was designated as *sd1-bm* based on its source (Bindli mutant).

### 2.5. dCAPS Marker Validation of Causal SNP

**Figure 3:**
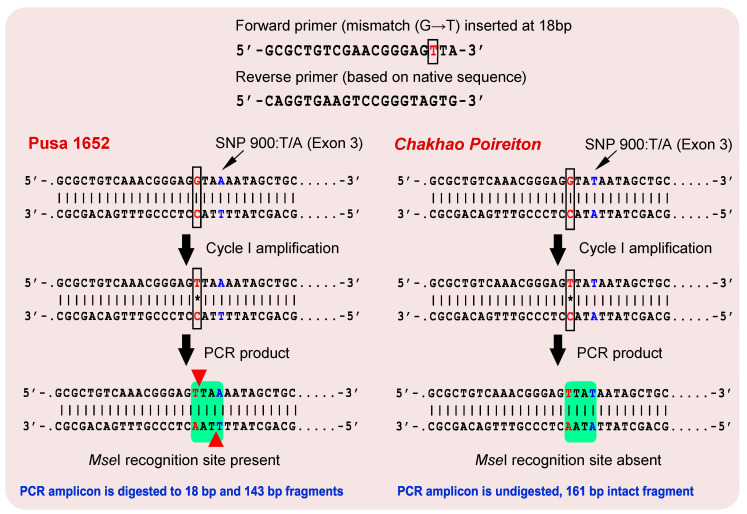
Development of derived cleaved amplified polymorphic sequence (dCAPS) marker, AKS-Sd1, and the validation of causal single-nucleotide polymorphism (SNP). (A) A restriction site for *Mse*I was generated, including SNP, which is cut by the enzyme producing cleaved products in Pusa 1652, while no cleaving occurs in *Chakhao Poireiton*.

**Figure 4:**
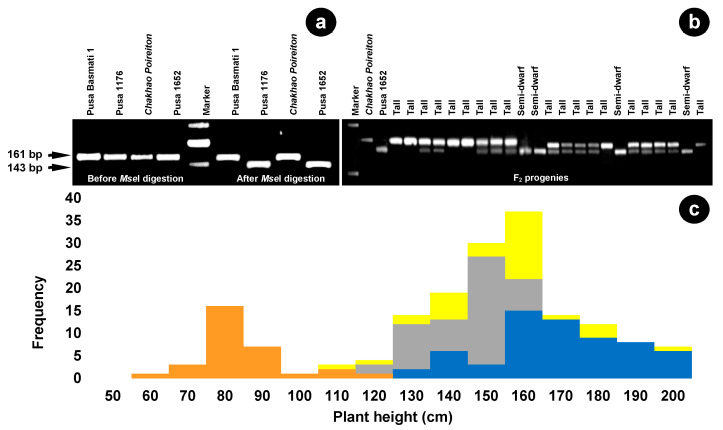
Gel pictogram of the dCAPS marker, AKS-sd1, (**a**) showing the clear differentiation of amplified and cleaved fragments in Pusa 1652 and its parent, Pusa 1176, which carries the novel sd1-bm allele. (**b**) A representative gel image of the parents and the F_2_ segregants showing the differentiation of height classes. (**c**) Frequency distribution of plant height among the different allelic classes of AKS-sd1, perfectly matched to the bimodal distribution. Segregants carrying the Pusa 1652 allele (*sd1-bm*) are shown in orange, and those with the *Chakhao Poireiton* allele (*SD1*) are shown as blue bars. The heterozygotes are shown as grey bars. The yellow bars indicate the overall frequency distribution of F_2_ plants.

A derived cleaved amplified polymorphic sequence (dCAPS) marker, AKS-sd1, was designed to target the first SNP of exon 3 in the *sd1-bm* allele with a forward primer, 5′-gcgctgtcgaacgggagtta-3′ that had an introduced recognition site (TTAA) for the *Mse*I restriction enzyme. The reverse primer was 5′-caggtgaagtccgggtagtg-3′. Amplification of the target region using AKS-sd1 resulted in a 161-bp fragment in both *Chakhao Poireiton* and Pusa 1652 (Figure 3). Restricted digestion of the amplicon with the *Mse*I enzyme resulted in two fragments in Pusa 1652, with fragment sizes of 143 bp and 18 bp, whereas in *Chakhao Poireiton* no digestion was observed and the amplicon remained intact at 161 bp (Figure 4a). The dCAPS marker was then validated in a set of 176 F_2_ plants which showed perfect co-segregation with the plant height phenotype (Figure 4b), wherein 48 tall plants were homozygous with the tall allele (161 bp fragment), 89 tall plants were heterozygous with the 161 bp and 143 bp fragments and 39 plants were homozygous with the semi-dwarf allele (143 bp). Furthermore, the frequency distribution of plant height among the segregants that are homozygous with the *sd1-bm* allele (Pusa 1652) showed a perfect fit for the semi-dwarf plants, while the tall class was found to be shared between homozygous *SD1* (*Chakhao Poireiton* allele) carriers and heterozygotes, making it evident that this SNP is the causal mutation for semi-dwarfism in Pusa 1652 (Figure 4c).

### 2.6. Agronomic Effect of sd1-bm Allele

Some of the backcross-derived lines (BLs) from the cross *Chakhao Poireiton*/Pusa 1652, after two backcrosses and one selfing, gained a recurrent genome recovery of more than 87.5% (Table 2; Figure S5). Furthermore, a comparison of agronomic data of those BLs indicated a significant reduction in plant height from that of the recurrent parent, *Chakhao Poireiton*. While the number of productive tillers in the BLs was comparable to that of the donor parent, Pusa 1652, it was two to three times more than that of *Chakhao Poireiton*. However, there was a slight reduction in panicle length in the BLs relative to *Chakhao Poireiton* (Table 2). 

## 3. Discussion

The semi-dwarf trait is recognized as one of the most preferred agronomic traits in rice, because of the ability to improve yields through higher harvest index, better nitrogen response, lodging resistance and better photosynthetic efficiency [13]. In rice, the *SD1* gene located at 38.38 Mb on the long arm of chromosome 1 encoding for GA20 oxidase 2 (*OsGA20ox2*) has been reported to control the semi-dwarf trait. Later, multiple mutant alleles of *SD1* have been identified, resulting either from natural or induced mutations. A few of these alleles have been utilized in varietal development [14]. The semi-dwarf trait conditioned by these *sd1* alleles is recessive to the tall phenotype produced by the wild type allele, *SD1*. Among the subspecies of rice, *indica* cultivars are generally taller than *japonica* cultivars [15]. This height difference between the sub-species was shown to be due to two non-synonymous SNPs in the *SD1* gene, present on exon 1 at the 299th nucleotide position (A in *japonica* and G in *indica*) and at the 1099th nucleotide position on exon 3 (A in *japonica* and G in *indica*) [16]. Based on the whole genome sequence, the *japonica* cultivar, Nipponbare was found to possess this wild *japonica* allele. The *Chakhao Poireiton* used in this study also has the same wild *japonica* allele. However, the allele in Pusa 1652 was similar to that of wild *indica* subtypes. When introgressed, both the wild *indica* and *japonica SD1* alleles were demonstrated to increase the plant height of IR36, an *indica* cultivar possessing the *sd1-d* allele [16]. The possession of the *japonica SD1* allele in *Chakhao Poireiton*, a tall rice cultivar popularly known as Manipur black rice [17], could also draw support for the fact that several of the rice cultivars of hill districts of northeast India have *japonica* lineages [18]. Although naturalized, the traditional rice cultivars of the eastern Himalayan region, which comprises Northeast India, contain 62.5% *indica* and 37.5% *japonica* species [19]. This naturalization process has made them genetic admixtures of subspecific populations [20].

Because of the extremely low frequency of useful allelic variants within *indica* group, *sd1-d* gained popularity as a ‘strong allele’ and is the only allele that has found its way into the majority of the modern high-yielding green revolution cultivars. While *sd1-d* had a loss of function mutation in *GA20ox2* activity through a deletion, the *SD1* mutant alleles of the *japonica* group were mostly associated with distinct SNPs. However, these SNPs either caused amino acid substitutions or nonsense mutations, resulting in the truncation of GA20ox2. For instance, in the *japonica* cultivar Calrose 76, a C→T shift in exon 2 at the 266th amino acid resulted in a substitution of leucine to phenylalanine, leading to a non-functional protein. In another *japonica* cultivar, Zhayeqing 8, an SNP in exon 2 caused a proline to leucine substitution at the 240th amino acid. Similarly, in the first semi-dwarf variety in China, Aijio-Nante, a 2 bp deletion in exon 1 induced a frameshift mutation, creating a stop codon [14]. Furthermore, two other mutants, due to a base substitution in exon 3, have been reported, one in the popular Chinese variety 9311, in which the 342nd amino acid, tyrosine, was changed to a stop codon. In another cultivar, Reimei, a G→C substitution in exon 3 caused a shift from aspartic acid to histidine at the 349th amino acid position, resulting in a non-functional polypeptide [8,14,21]. Several of these alleles are relatively ‘milder’ in effecting plant height reduction [12] and are found to be distributed in medium–tall *japonica* cultivars. For instance, a G→T substitution mutation in exon 1 at position 38382746 bp, causing the substitution of glycine to valine, detected in a Jukkoku mutant, has been identified in several other *japonica* cultivars such as Hikarishinseiki, Nishihomare, Hanasatsuma, Minamihikari, Reihou, Saiwaimochi, Hiyokumochi, Ayanatsuki, Yumehikari, Shironui and Yumehayato [22].

In rice, there have been several attempts to identify new genes for semi-dwarfism/dwarfism as well as different alleles of *SD1* through both induced and spontaneous mutations. Dominant or semi-dominant rice mutants obtained through chemical mutagenesis, such as KL908 carrying the *D53* gene [23], DMF-1 with the *Ssi1* gene [24], Y98149 with *Sdd(t)* [25], *Twisted dwarf 1-1* (*Tid1-1*) encoding the α-tubulin protein [26], LB4D carrying the *LB4D* gene [27], *Slr1-d* with a gain of function in the SLR1 protein [3,28], HD1 carrying the *d-h* gene [29], *Bdt1* obtained by DNA transposon [30], SV14 with the *Shortened Basal Internodes* (*SBI*) gene on chromosome 5 [13], were reported in *japonica* genotypes. The semi-dominant quantitative trait loci (QTL), *qDH1*, was identified on chromosome 1 in upland rice landrace, Kaowenghan [31], while, in *indica*, a recessive gene, *sd-c*, located at the centromeric region of chromosome 12 has been reported in *indica* cv. 93-11 through spontaneous mutation [32].

In the present study, we found that the semi-dwarf genotype, Pusa 1652 did not carry the *sd1-d* allele when tested using the functional marker based on the characteristic 383bp deletion, but instead amplified a fragment of 731 bp, which was similar to tall genotypes. This prompted us to look for the causal mutation in Pusa 1652. As Pusa 1652 is an improved semi-dwarf version of a short-grain aromatic landrace, Kalanamak, obtained by crossing with Pusa 1176, the semi-dwarfism in Pusa 1652 (Pusa 1176/Kalanamak//Kalanamak*1) could be traced back to Pusa 1176 based on pedigree data (Figure S4). Pusa 1176 is an aromatic semi-dwarf variety, but Kalanamak is a tall landrace [33]. Furthermore, Pusa 1176 has been developed from a cross between a mutant, Bindli Mutant 34 (BM34), and an aromatic landrace from Assam, IRGC16136. BM34 stands for Bindli Mutant 34, a semi-dwarf γ-ray induced mutant originated from the tall short-grain aromatic landrace, Bindli [34]. Having confirmed the monogenic inheritance of a semi-dwarf habit in Pusa 1652, we could further identify that plant height in this genotype showed a similar GA_3_ response to that of IR64, a known *sd1-d* carrier.

To assess whether the endogenous GA_3_ reduction was due to a mutation in the *SD1* gene, the marker based analysis in the genomic region of the *SD1* revealed that one of the linked markers, RM472 co-segregated with the semi-dwarf stature. By subsequent linkage analysis, RM472 was found to be linked at a distance of 8.7 cM from the gene, suggesting that *SD1* gene is responsible for determining semi-dwarfism in Pusa 1652. Furthermore, a comparison of the *SD1* gene sequence in Pusa 1652, with Nipponbare and *Chakhao Poireiton*, could help identify four SNPs, one on exon 1 and three on exon 3. Among these, the first SNP on exon 1 and the third on exon 3 were already known to be the causal SNPs for plant height difference between *indica* and *japonica* genotypes [16]. This drew our interest towards the remaining two SNPs on exon 3, which were unreported. We found that one of the novel SNPs on exon 3, the first among the three detected, had a transversion that led to a stop codon, rendering a truncated translation product. This caused a terminal polypeptide truncation—only eight amino acids were added instead of 98 amino acids from exon 3 (Figure 3)—which was responsible for reducing the plant height without causing the abnormal phenotypic effect. The subsequent non-synonymous SNPs identified on exon 3, were inconsequential, since they were preceded by the stop codon. Thus, the *sd1-bm* allele reported here is established to be different from the earlier reported alleles of *indica* rice types, as well as of *japonica* types such as in Jukkoku, Calrose 76, Zhayeqing 8, Reimei and 9311. However, to delineate the exact agronomic effect of *sd1-bm*, it would be worthwhile developing near isogenic lines (NILs) possessing different *SD1* alleles with a common background, so that their effectiveness in rice breeding could be underlined.

Nevertheless, *sd1-bm* offers a potential alternative to *sd1-d*, as evidenced by the agronomic performance of Pusa 1176, a popular aromatic cultivar of eastern India, which was not reported to have any agronomic disabilities (data not shown). Its derivative, Pusa 1652, a distinctly improved high-yielding version of Kalanamak, also does not show any adverse effects on growth and productivity attributed to the *sd1-bm* allele. Pusa 1652 possesses a reduced height coupled with a high yield and similar grain quality to that of Kalanamak. Similar agronomic properties are also observed among the BLs derived from *Chakhao Poireiton* (unpublished data). Additionally, to aid marker-assisted transfer, a functional dCAPS marker, AKS-sd1, was demonstrated to show perfect co-segregation with the semi-dwarfism in this study. This functional marker can help in the marker-assisted introgression of the semi-dwarfism trait in rice using Pusa 1652 or Pusa 1176 as a donor. 

## 4. Materials and Methods

### 4.1. Plant Materials

Pusa 1652 is a high-yielding semi-dwarf short-grain aromatic rice genotype developed at ICAR—Indian Agricultural Research Institute (ICAR—IARI), New Delhi, from a cross between a short-grain aromatic semi-dwarf genotype, Pusa 1176, and a tall popular short-grain aromatic rice landrace, *Kalanamak*. A cross was made between Pusa 1652 with *Chakhao Poireiton,* a tall Manipur black rice landrace during the *Kharif* season (June–October) in 2017 in New Delhi. The F_1_ was grown at the IARI Rice Breeding and Genetics Research Centre (RBGRC), Aduthurai, during the ensuing *Rabi* season (Nov 2018–Apr 2018). Selected F_1_s were selfed to produce F_2_ generation. The parents, F_1_ and the F_2_ population were raised during the subsequent *Kharif* season in 2018 in New Delhi. All the materials were grown under transplanted field conditions and managed under irrigated ecology with recommended agronomic practices.

### 4.2. Analysis of Inheritance of Plant Height

A genetic analysis of semi-dwarfism in Pusa 1652, was carried out using the parents, F_1_ and the F_2_ population from the cross Pusa 1652/*Chakhao Poireiton*. During *Kharif* 2018, plant height was recorded in the parents, F_1_s and 315 F_2_ plants at grain maturity, following the standard evaluation system of rice [35]. Plants were initially classified as tall (>130 cm), intermediate (110–130 cm) and semi-dwarf (<110 cm). The frequency distribution of the plant height among the F_2_ segregants was also compared graphically and the tall and intermediate classes were merged into the tall class based on the frequency distribution. Segregation analysis was carried out using the chi-square test for goodness of fit. 

### 4.3. GA_3_ Response in the Seedling Stage

To determine whether the semi-dwarf phenotype of Pusa 1652 was due to the insufficiency of endogenous GA_3_ production, the seedlings of both Pusa 1652 and IR64 (a semi-dwarf check with an *sd1-d* allele) along with *Chakhao Poireiton* (the tall check) were sprayed with 100 ppm of GA_3_ [36] when the seedlings were 10 days old and were at the two-leaf stage. For this, each genotype was sown in six pots filled with vermiculite mixture. A total of 20 seeds were sown equidistantly in every pot. Ten days after sowing, five uniform-looking seedlings were individually tagged from each pot and the initial seedling height was measured. The six pots were then divided into two sets of three pots each. One set received GA_3_ treatment and the other set was sprayed with the blank. For spraying, GA_3_ solution was prepared by initially dissolving 100 mg of GA_3_ in 5 mL of ethanol and making up the volume to 1000 mL. The blank was prepared similarly but without GA_3_. The spraying of GA_3_ solution as well as the blank was done on all the seedlings in corresponding pots. One week after the treatment, the plant height was measured from the same tagged seedlings in both control and treated pots. The standardized height increase was worked out as the ratio of height difference after GA_3_ treatment between sprayed and unsprayed seedlings to the standard average height before GA_3_ application.

### 4.4. Molecular Analysis and Mapping of the Semi-Dwarfing Gene

For carrying out molecular analysis, the genomic DNA was extracted from the test plants following the cetyl trimethyl ammonium bromide (CTAB) method [37]. Initially, Pusa 1652 was checked for the presence of the *sd1-d* allele, along with its parent, Pusa 1176, and two other semi-dwarf genotypes, Pusa Basmati 1 and IR64, using the marker *SD1* (F: 5′-cacgcacgggttcttccaggtg-3′, R: 5′-aggagaataggagatggtttacc-3′) which targets the 383 bp deletion [38]. Genotypes such as Nagina 22 and *Chakhao Poireiton* were used as the tall checks carrying the wild *SD1* allele. Subsequently, Pusa 1652 was subjected to a polymorphism survey against *Chakhao Poireiton*, using 12 SSR markers from the *SD1* genomic region on chromosome 1, sourced from the genetic map of rice [39]. A polymerase chain reaction (PCR) was carried out in reaction mixture with a total volume of 10 µl, constituted with 5 µl of 2X PCR master mix (Genei^TM^, Bangalore, India), 1 µl of 5 pmol of forward and reverse primer each and 1.2 µl of 20–40 ng DNA. The PCR was carried out with the following program: an initial denaturation period of 5 min at 95 °C, followed by a thermal profile consisting of 35 cycles of 30s at 95 °C, 30s at a particular annealing temperature and 1 min at 72 °C, followed by a final extension at 72 °C for 10 min. The amplified PCR product was resolved on 3.5% agarose gel stained with ethidium bromide run in 1X TAE buffer and the bands were visualized in a gel documentation system (Bio-Rad Laboratories Inc., Hercules, USA).

Bulked segregant analysis (BSA) [40] was adopted for identifying putatively linked marker(s) associated with the gene governing the semi-dwarf trait in Pusa 1652 using the markers that were polymorphic between the parents. Prior to this, DNA bulks were constituted by mixing an equimolar concentration of genomic DNA from 10 individual F_2_ plants exhibiting contrasting plant heights, one for the tall class and the other for the semi-dwarf class. The tall and semi-dwarf bulks, along with the parents, Pusa 1652 and *Chakhao Poireiton*, were genotyped with the polymorphic markers. The markers that distinguished the bulks were identified as putatively linked to the height classes. One of the putatively linked marker(s) that was physically closer to *SD1* (RM472) was further used for genotyping all the 315 F_2_ plants for which plant height was recorded. Linkage analysis between the putatively linked marker and the plant height was carried out using MAPMAKER/EXP 3.0 [41].

### 4.5. Identification of the Functional SNP in Pusa 1652

The data on the genome sequences of Pusa 1652 and *Chakhao Poireiton* were generated on Illumina Hiseq 2500 at a depth of 50X coverage. The sequences were aligned to the reference genome of Nipponbare as per standard procedures using the programs Burrows–Wheeler Aligner (BWA) [42] and Sequence Alignment/Map (SAM) tools [43]. The variants in the locus LOC_Os01g66100 (38382382 to 38385504 bp) were extracted and compared. The putative causal SNP was also reconfirmed through amplicon sequencing from Pusa 1652 and *Chakhao Poireiton* using an ABI 3730 XL DNA Analyzer with a BigDye^®^ terminator v3.1 cycle sequencing kit (Applied Biosystems Inc., Foster City, CA, USA). The primer pair was designed using Primer 3.0 software [44] and the amplicon sequences were compared using sequence alignment editor software BioEdit v.7.2.

### 4.6. Development and Validation of dCAPS Markers Based on Causal SNP in sd1-bm

To validate the causal SNP determining semi-dwarfism in Pusa 1652, a derived cleaved amplified polymorphic sequence (dCAPS) marker was designed [45] using dCAPS Finder 2.0. The two identical sequences with an SNP in the middle, with approximately 25 nucleotides on each side, were entered into dCAPS Finder 2.0. The nucleotides of *Chakhao Poireiton* and Pusa 1652 at the SNP position were entered as wild and mutant sequences, respectively. If a CAPS marker was not generated with zero mismatches, one mismatch was entered to search for a dCAPS marker. One mismatch produced several optional primer outputs for both the wild type and the mutant sequences. One primer that included a mismatch in the 3′ end close to the SNP, which also created a recognition site for a restriction enzyme, was chosen as the forward primer. The reverse primer was designed using the original sequence covering the target SNP, and had a similar GC content as that of the forward primer. The primers were designed with the aid of the Primer-BLAST tool. Two primer sequences were designed separately, covering either position of the identified SNP that formed the final primer pair of dCAPS for *MSe*I. The aliquots (5 µL) of the PCR product were incubated at 37 °C for 45 min with 2 µL of 10X restriction buffer and 1 µL of *Mse*I (10 U/µL) restriction enzyme (NEB, R0525L) in a total volume of 10 µL. The final digested product was resolved in 3.5% agarose gel stained with ethidium bromide.

### 4.7. Agronomic Effect of sd1-bm Allele

To test the agronomic effect of the *sd1-bm* allele, the backcross-derived lines (BC_2_F_2_) from the cross *Chakhao Poireiton*/Pusa 1652 were generated through two backcrosses with *Chakhao Poireiton* followed by selfing. The backcross-derived lines (BLs) were assessed for the recovery of recurrent parent genome (RPG), computed using the formula, RPG (%) = (R + 0.5H) × 100/P, where R is the total number of homozygous alleles belonging to the recurrent parent, H is the total number of heterozygous markers and P is the total number of polymorphic markers between the parents [46]. Later, the semi-dwarf progenies with the highest RPG recovery were evaluated in New Delhi along with their parents for agronomic traits such as plant height at maturity in centimeters (PHt), number of productive tillers (NPT), and panicle length in centimeters (PnL).

## 5. Conclusions

To conclude, we discovered a novel allele, *sd1-bm* of the *SD1* gene, governing the semi-dwarf trait in Pusa 1652 in the present study. Originally sourced from BM34, a γ-ray induced mutant of Bindli, *sd1-bm*, results in the semi-dwarfism attributed to a truncated product of the *OsGA20ox2* gene, due to a nonsense mutation in exon 3, resulting in a premature stop codon at the 300th amino acid in the place of tyrosine. The dCAPS marker, AKS-sd1, developed based on this functional SNP, can help in marker-assisted introgression of this novel allele for the semi-dwarf trait in rice, thereby diversifying the semi-dwarfism source in rice cultivar development.

## Figures and Tables

**Table 1:** Response of plant height of genotypes Pusa 1652, *Chakhao Poireiton* and IR64 to external application of gibberellic acid (GA_3_) at seedling stage.

Genotype	Control		GA_3_ Treated		Relative Response in Plant Height (%)
	PH_10_	PH_18_	PH_10_	PH_18_	
Pusa 1652	7.5 ^c^	18.7 ^c^	7.3 ^c^	27.1 ^c^	45.3 ^a^
*Chakhao Poireiton*	14.0 ^a^	30.8 ^a^	14.8 ^a^	37.7 ^a^	22.4 ^b^
IR64 (*sd1-d* check)	13.1 ^b^	21.8 ^b^	13.9 ^b^	30.9 ^b^	41.4 ^a^
CD (*p* < 0.05)	0.64	0.40	0.33	2.03	12.9

PH_10_ and PH_18_, plant height in centimeters, respectively, 10 and 18 days after sowing; relative response to percentage of GA_3_ application. Means followed by similar letters are statistically not significant at *p* < 0.05 based on the least significant difference (LSD) test. The critical difference (CD) is 0.05 at the 5% probability level, based on the LSD test.

**Table 2:** Agronomic data of backcross-derived lines of *Chakhao Poireiton*/Pusa 1652 showing the height reduction and increased number of productive tillers.

Genotype	PHt	NPT	PnL	RPG (%)
BL 1	87.0 ^b^	16.0 ^a^	24.0 ^ab^	87.5
BL 2	91.0 ^b^	17.0 ^a^	20.0 ^b^	87.5
BL 3	103.0 ^b^	14.0 ^a^	25.0 ^ab^	91.6
BL 4	97.0 ^b^	18.0 ^a^	21.0 ^b^	91.6
*Chakhao Poireiton*	153.2 ^a^	6.4 ^b^	28.0 ^a^	-
Pusa 1652	87.6 ^b^	14.0 ^a^	19.8 ^b^	-
CD (*p* < 0.05)	16.81	4.22	5.44	-

PHt—plant height at maturity in centimeters; NPT—number of productive tillers; PnL—panicle length in centimeters; RPG—recurrent parent genome recovery in percentage. The means followed by the same letters are statistically not different based on the least significant difference (LSD) test.

## Supplementary Materials

The following are available online at https://www.mdpi.com/2223-7747/9/9/1198/s1, Figure S1: Responses of IR64, Pusa 1652 and *Chakhao Poireiton* for exogenous application of GA_3_ at the seedling stage; increase in height induced by the GA_3_ in Pusa 1652 was comparable to the height increase in IR64, which carries the *sd1-d* allele, Figure S2: Linkage map of markers used for polymorphism survey between Pusa 1652 and *Chakhao Poireiton* on chromosome 1. Markers that were found polymorphic between parents are denoted in bold. The *SD1* locus is marked red. Three markers in blue, showed clear polymorphism between the bulks, Figure S3: Coding sequence (CDS) comparison of *SD1* gene between Nipponbare, *Chakhao Poireiton* and Pusa 1652, showing the single nucleotide mutations at positions 299, 900, 903 and 1019, Figure S4: Pedigree of Pusa 1652, Table S1: SNPs identified between Pusa 1652 and *Chakhao Poireiton*, and the associated amino acid change in *SD1* (LOC_Os01g66100) locus.
