# Supplementary material for: Discovery of a Novel Induced Polymorphism in SD1 Gene Governing Semi-Dwarfism in Rice and Development of a Functional Marker for Marker-Assisted Selection
Source: Plants (Basel). 2020 Sep 14;9(9):1198. doi: 10.3390/plants9091198 (PMC7570060; doi:10.3390/plants9091198)
Supplement: Supplementary file 1 [file plants-09-01198-s001.pdf]

Supplementary information

Discovery of a novel induced polymorphism in *SD1* gene governing semi-dwarfism in rice and development of a functional marker for marker-assisted selection

Shivashankar Bhuvaneswari, Subbaiyan Gopala Krishnan, Ranjith Kumar Ellur, Kunnummal Kurungara Vinod, Haritha Bollinedi, Prolay Kumar Bhowmick, Vijay Prakash Bansal, Mariappan Nagarajan and Ashok Kumar Singh

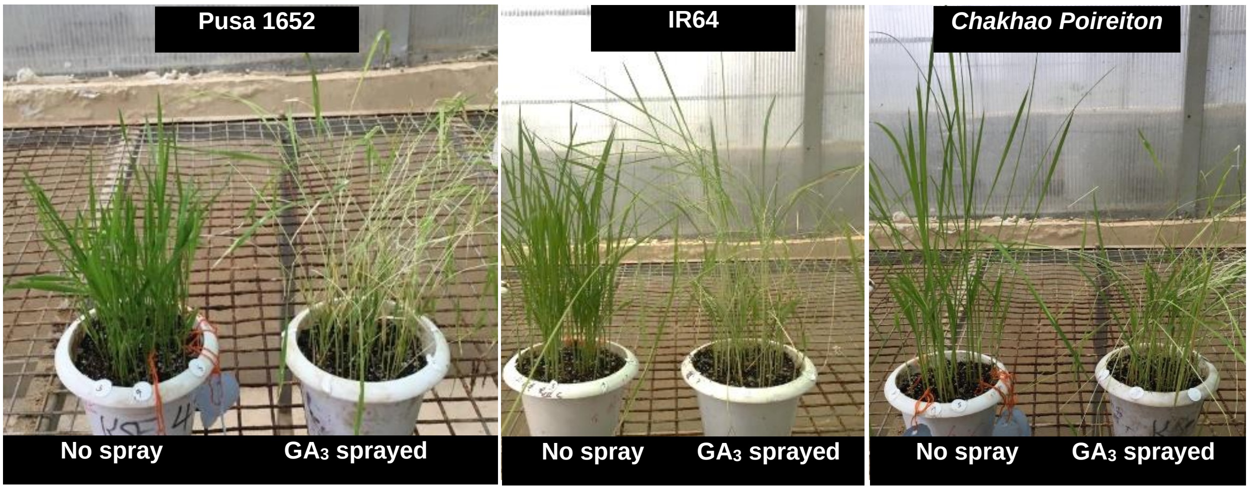

Figure S1. Responses of IR64, Pusa 1652 and *Chakhao Poireiton* for exogenous application of GA_3 at the seedling stage; increase in height induced by the GA_3 in Pusa 1652 was comparable to the height increase in IR64, which carries the *sd1-d* allele.

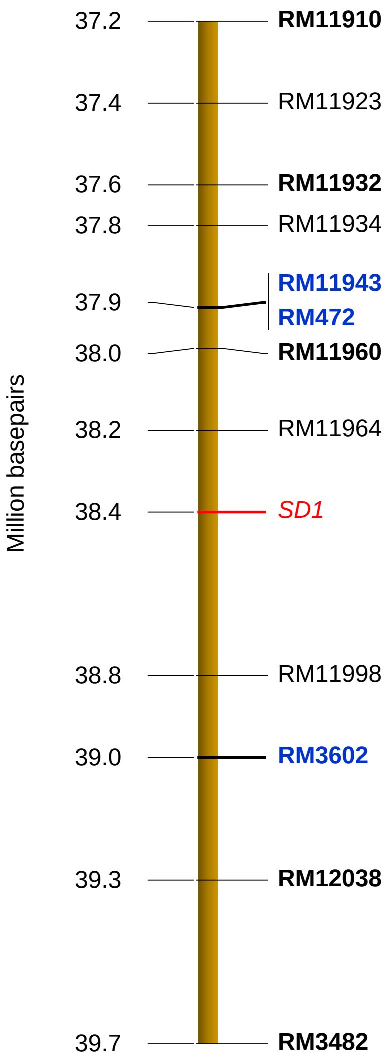

Figure S2. Linkage map of markers used for polymorphism survey between Pusa 1652 and *Chakhao Poireiton* on chromosome 1. Markers that were found polymorphic between parents are denoted in bold. The *SD1* locus is marked red. Three markers in blue, showed clear polymorphism between the bulks.

Nipponbare Genome IRGSP 1.0 Release 7.0 LOC_Os01g66100.1 (38382382-38385504)

```
SD1|Nipponbare          ATGGTGGCCGAGCACCCCACGCCACCACAGCCGCACCAACCACCGCCCATGGACTCCACC 60
SD1|Chakhao_Poireiton   ATGGTGGCCGAGCACCCCACGCCACCACAGCCGCACCAACCACCGCCCATGGACTCCACC
SD1|PUSA_1652           ATGGTGGCCGAGCACCCCACGCCACCACAGCCGCACCAACCACCGCCCATGGACTCCACC

SD1|Nipponbare          GCCGGCTCTGGCATTGCCGCCCCGGCGGCGGCGGCGGTGTGCGACCTGAGGATGGAGCCC 120
SD1|Chakhao_Poireiton   GCCGGCTCTGGCATTGCCGCCCCGGCGGCGGCGGCGGTGTGCGACCTGAGGATGGAGCCC
SD1|PUSA_1652           GCCGGCTCTGGCATTGCCGCCCCGGCGGCGGCGGCGGTGTGCGACCTGAGGATGGAGCCC

SD1|Nipponbare          AAGATCCCGGAGCCATTCGTGTGGCCGAACGGCGACGCGAGGCCGGCGTCGGCGGCGGAG 180
SD1|Chakhao_Poireiton   AAGATCCCGGAGCCATTCGTGTGGCCGAACGGCGACGCGAGGCCGGCGTCGGCGGCGGAG
SD1|PUSA_1652           AAGATCCCGGAGCCATTCGTGTGGCCGAACGGCGACGCGAGGCCGGCGTCGGCGGCGGAG

SD1|Nipponbare          CTGGACATGCCCGTGGTCGACGTGGGCGTGCTCCGCGACGGCGACGCCGAGGGGCTGCGC 240
SD1|Chakhao_Poireiton   CTGGACATGCCCGTGGTCGACGTGGGCGTGCTCCGCGACGGCGACGCCGAGGGGCTGCGC
SD1|PUSA_1652           CTGGACATGCCCGTGGTCGACGTGGGCGTGCTCCGCGACGGCGACGCCGAGGGGCTGCGC

SD1|Nipponbare          CGCGCCGCGGCGCAGGTGGCCGCCGCGTGCGCCACGCACGGGTTCTTCCAGGTGTCCGAG 300
SD1|Chakhao_Poireiton   CGCGCCGCGGCGCAGGTGGCCGCCGCGTGCGCCACGCACGGGTTCTTCCAGGTGTCCGAG
SD1|PUSA_1652           CGCGCCGCGGCGCAGGTGGCCGCCGCGTGCGCCACGCACGGGTTCTTCCAGGTGTCCGGG

SD1|Nipponbare          CACGGCGTCGACGCCGCTCTGGCGCGCGCCGCGCTCGACGGCGCCAGCGACTTCTTCCGC 360
SD1|Chakhao_Poireiton   CACGGCGTCGACGCCGCTCTGGCGCGCGCCGCGCTCGACGGCGCCAGCGACTTCTTCCGC
SD1|PUSA_1652           CACGGCGTCGACGCCGCTCTGGCGCGCGCCGCGCTCGACGGCGCCAGCGACTTCTTCCGC

SD1|Nipponbare          CTCCCGCTCGCCGAGAAGCGCCGCGCGCGCCGCGTCCCGGGCACCGTGTCCGGCTACACC 420
SD1|Chakhao_Poireiton   CTCCCGCTCGCCGAGAAGCGCCGCGCGCGCCGCGTCCCGGGCACCGTGTCCGGCTACACC
SD1|PUSA_1652           CTCCCGCTCGCCGAGAAGCGCCGCGCGCGCCGCGTCCCGGGCACCGTGTCCGGCTACACC

SD1|Nipponbare          AGCGCCCACGCCGACCGCTTCGCCTCCAAGCTCCCATGGAAGGAGACCCTCTCCTTCGGC 480
SD1|Chakhao_Poireiton   AGCGCCCACGCCGACCGCTTCGCCTCCAAGCTCCCATGGAAGGAGACCCTCTCCTTCGGC
SD1|PUSA_1652           AGCGCCCACGCCGACCGCTTCGCCTCCAAGCTCCCATGGAAGGAGACCCTCTCCTTCGGC

SD1|Nipponbare          TTCCACGACCGCGCCGCCGCCCCCGTCGTCGCCGACTACTTCTCCAGCACCCTCGGCCCC 540
SD1|Chakhao_Poireiton   TTCCACGACCGCGCCGCCGCCCCCGTCGTCGCCGACTACTTCTCCAGCACCCTCGGCCCC
SD1|PUSA_1652           TTCCACGACCGCGCCGCCGCCCCCGTCGTCGCCGACTACTTCTCCAGCACCCTCGGCCCC

SD1|Nipponbare          GACTTCGCGCCAATGGGGAGGGTGTACCAGAAGTACTGCGAGGAGATGAAGGAGCTGTCG 600
SD1|Chakhao_Poireiton   GACTTCGCGCCAATGGGGAGGGTGTACCAGAAGTACTGCGAGGAGATGAAGGAGCTGTCG
SD1|PUSA_1652           GACTTCGCGCCAATGGGGAGGGTGTACCAGAAGTACTGCGAGGAGATGAAGGAGCTGTCG

SD1|Nipponbare          CTGACGATCATGGAACTCCTGGAGCTGAGCCTGGGCGTGGAGCGAGGCTACTACAGGGAG 660
SD1|Chakhao_Poireiton   CTGACGATCATGGAACTCCTGGAGCTGAGCCTGGGCGTGGAGCGAGGCTACTACAGGGAG
SD1|PUSA_1652           CTGACGATCATGGAACTCCTGGAGCTGAGCCTGGGCGTGGAGCGAGGCTACTACAGGGAG

SD1|Nipponbare          TTCTTCGCGGACAGCAGCTCAATCATGCGGTGCAACTACTACCCGCCATGCCCGGAGCCG 720
SD1|Chakhao_Poireiton   TTCTTCGCGGACAGCAGCTCAATCATGCGGTGCAACTACTACCCGCCATGCCCGGAGCCG
SD1|PUSA_1652           TTCTTCGCGGACAGCAGCTCAATCATGCGGTGCAACTACTACCCGCCATGCCCGGAGCCG

SD1|Nipponbare          GAGCGGACGCTCGGCACGGGCCCGCACTGCGACCCCACCGCCCTCACCATCCTCCTCCAG 780
SD1|Chakhao_Poireiton   GAGCGGACGCTCGGCACGGGCCCGCACTGCGACCCCACCGCCCTCACCATCCTCCTCCAG
SD1|PUSA_1652           GAGCGGACGCTCGGCACGGGCCCGCACTGCGACCCCACCGCCCTCACCATCCTCCTCCAG

SD1|Nipponbare          GACGACGTCGGCGGCCTCGAGGTCCTCGTCGACGGCGAATGGCGCCCCGTCAGCCCCGTC 840
SD1|Chakhao_Poireiton   GACGACGTCGGCGGCCTCGAGGTCCTCGTCGACGGCGAATGGCGCCCCGTCAGCCCCGTC
SD1|PUSA_1652           GACGACGTCGGCGGCCTCGAGGTCCTCGTCGACGGCGAATGGCGCCCCGTCAGCCCCGTC

SD1|Nipponbare          CCCGGCGCCATGGTCATCAACATCGGCGACACCTTCATGGCGCTGTCGAACGGGAGGTAT 900
SD1|Chakhao_Poireiton   CCCGGCGCCATGGTCATCAACATCGGCGACACCTTCATGGCGCTGTCGAACGGGAGGTAT
SD1|PUSA_1652           CCCGGCGCCATGGTCATCAACATCGGCGACACCTTCATGGCGCTGTCGAACGGGAGGTAA

SD1|Nipponbare          AAGAGCTGCCTGCACAGGGCGGTGGTGAACCAGCGGCGGGAGCGGCGGTCGCTGGCGTTC 960
SD1|Chakhao_Poireiton   AAGAGCTGCCTGCACAGGGCGGTGGTGAACCAGCGGCGGGAGCGGCGGTCGCTGGCGTTC
SD1|PUSA_1652           AATAGCTGCCTGCACAGGGCGGTGGTGAACCAGCGGCGGGAGCGGCGGTCGCTGGCGTTC

SD1|Nipponbare          TTCCTGTGCCCGCGGGAGGACAGGGTGGTGCGGCCGCCGCCGAGCGCCGCCACGCCGCAG 1020
SD1|Chakhao_Poireiton   TTCCTGTGCCCGCGGGAGGACAGGGTGGTGCGGCCGCCGCCGAGCGCCGCCACGCCGCAG
SD1|PUSA_1652           TTCCTGTGCCCGCGGGAGGACAGGGTGGTGCGGCCGCCGCCGAGCGCCGCCACGCCGCGG

SD1|Nipponbare          CACTACCCGGACTTCACCTGGGCCGACCTCATGCGCTTCACGCAGCGCCACTACCGCGCC 1080
SD1|Chakhao_Poireiton   CACTACCCGGACTTCACCTGGGCCGACCTCATGCGCTTCACGCAGCGCCACTACCGCGCC
SD1|PUSA_1652           CACTACCCGGACTTCACCTGGGCCGACCTCATGCGCTTCACGCAGCGCCACTACCGCGCC

SD1|Nipponbare          GACACCCGCACGCTCGACGCCTTCACGCGCTGGCTCGCGCCGCCGGCCGCCGACGCCGCC 1140
SD1|Chakhao_Poireiton   GACACCCGCACGCTCGACGCCTTCACGCGCTGGCTCGCGCCGCCGGCCGCCGACGCCGCC
SD1|PUSA_1652           GACACCCGCACGCTCGACGCCTTCACGCGCTGGCTCGCGCCGCCGGCCGCCGACGCCGCC

SD1|Nipponbare          GCGACGGCGCAGGTCGAGGCGGCCAGCTGA.............................. 1170
SD1|Chakhao_Poireiton   GCGACGGCGCAGGTCGAGGCGGCCAGCTGA..............................
SD1|PUSA_1652           GCGACGGCGCAGGTCGAGGCGGCCAGCTGA..............................
```

Figure S3. Coding sequence (CDS) comparison of *SD1* gene between Nipponbare, *Chakhao Poireiton* and Pusa 1652, showing the single nucleotide mutations at positions 299, 900, 903 and 1019. Red arrow indicates the primer target sequences for the dCAPS marker, AKS-Sd1. The amplicon is marked within an irregular rectangle.

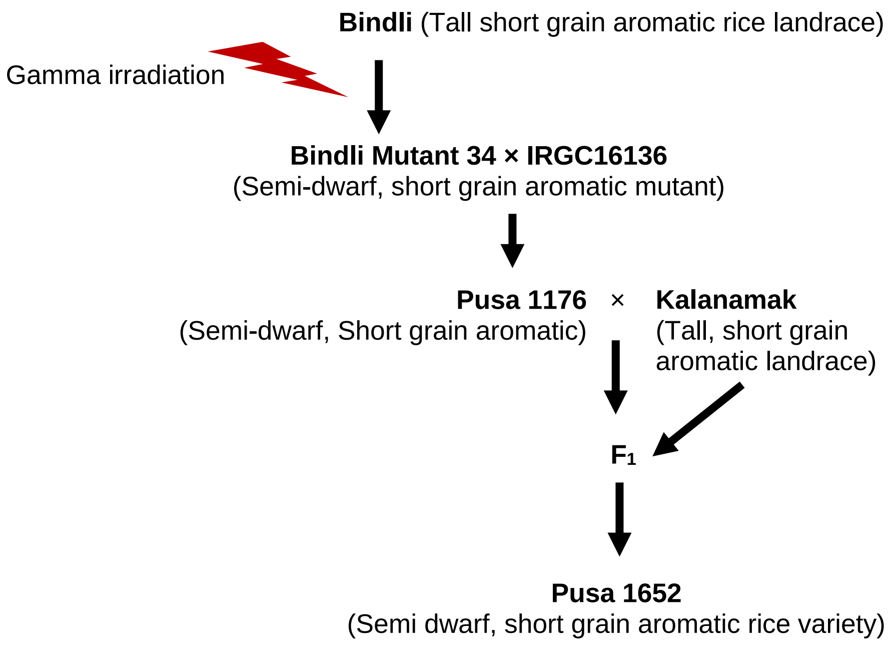

Figure S4. Pedigree of Pusa 1652

Table S1. SNPs identified between Pusa 1652 and *Chakhao Poireiton,* and the associated amino acid change in *SD1* (LOC_Os01g66100) locus

**Exon**	**Physical position**	**Nucleotide change**			**Position in polypeptide**	**Amino acid change**	
		**Nipponbare**	***C. Poireiton***	**Pusa 1652**		**Nipponbare/ *C. Poireiton***	**Pusa1652**
1	38382764	A	A	G	100	Glutamic acid (E)	Glycine (G)
**3**	**38384938**	**T**	**T**	**A**	**300**	**Tyrosine (Y)**	**STOP**
3	38384941	G	G	T	301	Lysine (K)	Aspargine (N)
3	38385057	A	A	G	340	Glutamine (Q)	Arginine (R)

C. Poireiton, Chakhao Poiretiton
